# Early sex differences are not autism-specific: A Baby Siblings Research Consortium (BSRC) study

**DOI:** 10.1186/s13229-015-0027-y

**Published:** 2015-06-04

**Authors:** Daniel S. Messinger, Gregory S. Young, Sara Jane Webb, Sally Ozonoff, Susan E. Bryson, Alice Carter, Leslie Carver, Tony Charman, Katarzyna Chawarska, Suzanne Curtin, Karen Dobkins, Irva Hertz-Picciotto, Ted Hutman, Jana M. Iverson, Rebecca Landa, Charles A. Nelson, Wendy L. Stone, Helen Tager-Flusberg, Lonnie Zwaigenbaum

**Affiliations:** University of Miami, Coral Gables, FL USA; University of California, Davis, FL USA; Seattle Children’s Research Institute, Seattle, WA USA; University of Washington, Seattle, WA USA; Izaak Walton Killam Health Centre, Dalhousie University, Halifax, NS Canada; University of Massachusetts, Boston, MA USA; University of California, San Diego, CA USA; King’s College London, London, UK; Yale University School of Medicine, New Haven, CT USA; University of Calgary, Calgary, AB Canada; University of California, Los Angeles, CA USA; University of Pittsburgh, Pittsburgh, PA USA; Kennedy Krieger Institute and John Hopkins School of Medicine, Baltimore, MD USA; Harvard Medical School, Boston, MA USA; Harvard Graduate School of Education, Cambridge, UK; Boston Children’s Hospital, Boston, MA USA; Boston University, Boston, MA USA; University of Alberta, Edmonton, AB Canada

**Keywords:** Sex differences, High-risk siblings, Symptom severity, Development, Longitudinal

## Abstract

**Background:**

The increased male prevalence of autism spectrum disorder (ASD) may be mirrored by the early emergence of sex differences in ASD symptoms and cognitive functioning. The female protective effect hypothesis posits that ASD recurrence and symptoms will be higher among relatives of female probands. This study examined sex differences and sex of proband differences in ASD outcome and in the development of ASD symptoms and cognitive functioning among the high-risk younger siblings of ASD probands and low-risk children.

**Methods:**

Prior to 18 months of age, 1824 infants (1241 high-risk siblings, 583 low-risk) from 15 sites were recruited. Hierarchical generalized linear model (HGLM) analyses of younger sibling and proband sex differences in ASD recurrence among high-risk siblings were followed by HGLM analyses of sex differences and group differences (high-risk ASD, high-risk non-ASD, and low-risk) on the Mullen Scales of Early Learning (MSEL) subscales (Expressive and Receptive Language, Fine Motor, and Visual Reception) at 18, 24, and 36 months and Autism Diagnostic Observation Schedule (ADOS) domain scores (social affect (SA) and restricted and repetitive behaviors (RRB)) at 24 and 36 months.

**Results:**

Of 1241 high-risk siblings, 252 had ASD outcomes. Male recurrence was 26.7 % and female recurrence 10.3 %, with a 3.18 odds ratio. The HR-ASD group had lower MSEL subscale scores and higher RRB and SA scores than the HR non-ASD group, which had lower MSEL subscale scores and higher RRB scores than the LR group. Regardless of group, males obtained lower MSEL subscale scores, and higher ADOS RRB scores, than females. There were, however, no significant interactions between sex and group on either the MSEL or ADOS. Proband sex did not affect ASD outcome, MSEL subscale, or ADOS domain scores.

**Conclusions:**

A 3.2:1 male:female odds ratio emerged among a large sample of prospectively followed high-risk siblings. Sex differences in cognitive performance and repetitive behaviors were apparent not only in high-risk children with ASD, but also in high-risk children without ASD and in low-risk children. Sex differences in young children with ASD do not appear to be ASD-specific but instead reflect typically occurring sex differences seen in children without ASD. Results did not support a female protective effect hypothesis.

**Electronic supplementary material:**

The online version of this article (doi:10.1186/s13229-015-0027-y) contains supplementary material, which is available to authorized users.

## Background

Robust elevations in the prevalence of autism spectrum disorder (ASD) among males relative to females may or may not be mirrored by sex differences in the emergence of ASD symptoms among boys and girls with ASD [1]. If present, sex differences in symptom presentation and cognitive functioning among children with ASD may be unique to the disorder or reflect normative sex differences present among children without ASD. Here we report on a large-scale prospective investigation of the high-risk younger siblings of ASD probands (and low-risk comparison children) to both address differential ASD occurrence and to characterize potential sex differences in the early ASD phenotype. These data afford a test of the female protective effect hypothesis, which proposes that the younger siblings of female probands will have higher odds of ASD recurrence and higher levels of ASD symptoms than the siblings of male probands.

ASD is more common in males than females [2], with an approximate 4:1 risk ratio estimate emerging both from literature review [3] and a school-based prevalence study of 8-year-olds [4]. However, recent community-based ascertainment initiatives have yielded ratios lower than 3:1 among Asian [5, 6] and European [7] children, and a non-significant male:female difference in a Swedish population cohort [8]. Prospective studies of high-risk infant siblings offer a view of the emergence of the ASD phenotype which may reduce the male ascertainment bias which has been documented in clinic-referred samples [9]. In high-risk sibling studies, enrollment typically occurs during infancy prior to the onset of symptoms, and outcome is ascertained at a fixed point, most often 3 years of age. Variable male:female ratios in prospectively followed high-risk sibling samples (2.8:1 [10] and 1.65:1 [11]) suggest the importance of large-scale characterization of the risk of ASD among high-risk siblings.

Sex differences may be present not only in ASD occurrence but in ASD symptoms and levels of cognitive functioning. Females with ASD have historically presented with lower IQ than boys [3, 12, 13]. Likewise, females diagnosed with ASD in the Simons Simplex Collection exhibited higher levels of social affect and communication symptoms on the Autism Diagnostic Observation Schedule (ADOS) than males with ASD, as well as lower verbal and nonverbal IQ [14]. However, a recent investigation did not reveal sex differences in cognitive performance or ASD symptom severity either among 3-year-olds with ASD or among typically developing children [15]. There is, in fact, evidence of greater ASD symptom severity—particularly elevated levels of repetitive and restricted behavior—among males. In both the Autism Genome Project [16] and a recent study of 3- and 4-year-olds [17], males with ASD had higher levels of repetitive behavior than females.

Recent studies on sex differences in the presentation of children with and without ASD also suggest greater symptom severity for males. A prospective investigation, for example, yielded some evidence of a female advantage (higher fine motor scores on the MSEL and lower ADOS severity scores) for high-risk siblings with an ASD outcome, high-risk siblings without an ASD outcome, and low-risk children [11]. There was no evidence that this sex difference varied by ASD outcome or risk status. Although not a common focus of developmental research, a substantial body of work on adults examines the possibility that ASD sex differences are a reflection of normative sex differences [18, 19]. These findings raise the possibility that sex differences seen in the ASD phenotype are not unique to ASD but reflect broader sex differences in the general population.

Sex differences in ASD occurrence may suggest a female protective effect. Clinically identified girls with ASD carry a higher load of deleterious genetic variants than boys [20] and may have a higher threshold for the impact of the multifactorial array of genetic and environmental factors thought to be responsible for ASD [21]. The female protective effect account hypothesizes that first-degree relatives of female probands will exhibit higher levels of ASD symptoms and higher levels of ASD recurrence than the first-degree relatives of male probands [21, 22]. Two reports indicate that the siblings of female probands present with higher levels of parent-reported ASD symptoms than the siblings of male probands [16, 22]. There is little evidence, however, that siblings of female probands exhibit a differential risk for the occurrence of categorical ASD [8, 21, 23–25].

Prospective studies of high-risk infant siblings offer a unique perspective on the role of younger sibling sex and proband sex in ASD occurrence and the emergence of the ASD phenotype. A previous report from the Baby Siblings Research Consortium (BSRC) utilizing slightly more than half (664) of the current sample of 1241 high-risk infants yielded an 18.7 % risk of ASD recurrence, which was elevated among males and among siblings from multiplex families [10]. In a subsequent BSRC report on non-diagnosed high-risk siblings [26], males in both high-risk (*n* = 507) and low-risk groups (*n* = 324) exhibited higher ASD symptom severity scores—and lower levels of verbal and nonverbal functioning—than females. No finer distinctions were made, however, in either ASD symptoms or cognitive functioning.

Here we report on younger sibling sex differences and proband sex differences on the odds of ASD in a large sample of prospectively followed high-risk siblings. Sex differences in ASD odds provide a context for examining younger sibling and proband sex differences in ASD-related social affect and repetitive behavior symptom severity, as well as multiple elements of cognitive functioning. Specifically, we investigate younger sibling and proband sex differences in the longitudinal development of symptom presentation and cognitive functioning among three groups of children: high-risk siblings with ASD, high-risk siblings without ASD, and low-risk children. We tested for sex and group differences in cognitive functioning and ASD symptom severity over age. We were particularly interested in ascertaining whether sex differences in symptom severity and cognitive functioning differed in these groups, which would be instantiated by a statistical interaction. The absence of such an interaction would suggest that male/female differences in symptom severity and cognitive functioning were not unique to ASD outcome or risk status, but instead reflected normative sex differences.

## Methods

### Participants

Data were pooled from 15 independently funded research sites that are part of the BSRC, an international network supported by Autism Speaks. The BSRC database is approved by the University of California Davis Institutional Review Board. Please see the “Acknowledgements” section for a list of all the review boards that approved the study. All sites used similar recruitment and sampling methodologies as well as standardized longitudinal diagnostic assessment procedures. Families were recruited from clinics and agencies serving individuals with ASD, community events, website and media announcements, fliers, mailings, and word-of-mouth. Across all sites, inclusion criteria for the high-risk infants involved diagnostic confirmation of ASD in probands, with no genetic or neurological conditions (e.g., fragile X, tuberous sclerosis) accounting for the ASD diagnosis. At each site, consent was provided by the parents or legal guardians of the infant participants and human subject’s approval was provided by the local university institutional review board.

All participants were identified as being either the full biological younger sibling of a proband with an ASD diagnosis (the high-risk group) or having no first-degree relatives with an ASD diagnosis (the low-risk group). Inclusion required enrollment prior to 18 months and ASD outcome categorization, which required both clinical diagnosis and meeting ADOS cut-off criteria for ASD. Inclusion in the profile analyses required ADOS and/or MSEL data at 18 and/or 24 months of age. Final ADOS and MSEL assessments were included in profile analyses if they occurred from 33–38 months. Within the LR group, there were six children (three males) with an ASD outcome; they were removed from analyses. The analysis data set contained 1824 infant participants, of whom 1241 were high-risk (HR) and 583 were low-risk (LR). Table 1 characterizes these three groups.

**Table 1:** Sample characteristics

	High-risk	High-risk	Low-risk
Variable	ASD (*n* = 252)	Non-ASD (*n* = 989)	Non-ASD (*n* = 583)
Sex (% male)	76.6	52.7	50.8
Age first seen (months)	7.62 (3.58)	7.39 (3.46)	6.85 (2.94)
Age at outcome (months)	37.13 (2.06)	36.97 (1.97)	36.97 (2.18)
Proband sex (% male)	80.7	83.8	–
Multiplex (%)	15.3	5.6	–
Non-Caucasian (%)	17.6	15.3	14.2
Maternal education (% H.S.)	11.9	7.1	4.0
Paternal education (% H.S.)	16.3	11.1	9.3

*H.S.* high school: schooling terminated at or before high school completion

### Measures

#### Clinical best estimate diagnosis

Clinical best estimate (CBE) diagnoses were made or verified by licensed clinicians when infants were between 33 and 49 months of age and were informed by ADOS scores, DSM-IV criteria, and cognitive and behavioral assessments. Clinical diagnoses were dichotomized into either ASD (including pervasive developmental disorder—not otherwise specified and autistic disorder) or non-ASD.

#### Autism Diagnostic Observation Schedule

The ADOS [27] is a standardized assessment of autism symptoms consisting of 25 to 30 items across four symptom domains: social interaction, communication, repetitive and stereotyped behaviors, and play. Items are scored as 0 (developmentally appropriate and not autistic), 1 (mildly atypical), 2 (atypical and autistic in quality), or 3 (severely autistic). The ADOS yields a total score and clinical cut-off scores for use in the diagnosis of ASD. The ADOS also provides severity scores in each of two symptom domains: 1) social affect (SA) involving communication and social interaction items, and 2) restricted and repetitive behavior (RRB) involving repetitive and stereotyped behavior items. These 10-point severity scores allow for examining change in symptom severity over time as they are calibrated across different ages and test versions [28]. Both the SA and RRB domain severity scores were used to investigate change in symptoms between 24 months and 36 months.

#### Mullen Scales of Early Learning

The Mullen Scales of Early Learning (MSEL) [29] is designed to assess four areas of functioning: fine motor, visual reception, expressive language, and receptive language. Age-equivalent scores on these four subscales were used to investigate developmental growth trajectories between 18, 24, and 36 months. Age-equivalent scores are calibrated in months and are more sensitive to the low performance common in ASD samples than standard scores [17, 30].

### Analysis plan

A first set of analyses examined recurrence rates of ASD outcome for males and female high-risk siblings. We employed a hierarchical generalized linear model (HGLM) wherein ASD outcome was treated as a dichotomous dependent variable. Predictor variables included high-risk sibling sex, proband sex, demographic variables such as maternal education, and multiplex status. To control for site differences in recurrence rates, site was included as a random effect.

We next tested for sex differences and sex by group interactions in cognitive functioning and ASD symptom severity. This second set of analyses modeled sex and group differences in the longitudinal trajectories of MSEL subscale scores and ASD severity scores. In these models, a group variable contrasted high-risk siblings with an ASD outcome, high-risk siblings without an ASD outcome, and low-risk children. These models involved profile analysis within the framework of HGLM where subscale/domain was treated as a repeated factor within each time point. This allowed for the simultaneous assessment and comparison of growth trajectories in each subscale/domain between by sex and group. Full factorial models were examined, which included all higher-order interactions between sex, group, subscale/domain, and age. Of critical importance to the current study, these models tested all two-way and higher-order interactions between sex and group.

The profile analysis for the MSEL included data from three ages—18, 24, and 36 months—which permitted modeling both random intercepts and slopes for each subject across subscales. The profile analysis model for the ADOS involved two ages—24 and 36 months—and thus age was considered a repeated factor. Additionally, for the ADOS, a negative binomial distribution with a log link was employed to approximate domain score distributions for analyses.

All analyses were conducted in *R* [31] using the *lme4* package [32]. All significance testing of model terms and parameters was conducted using denominator degrees of freedom calculated using a Satterthwaite approximation.

## Results

### Sex differences and recurrence rates of ASD

Analyses of recurrence rates in the high-risk (HR) sample consider the overall recurrence rate and the effects of proband sex, younger sibling sex, and multiplex status. The initial HGLM included only a random site effect, with no fixed effect predictors. Results revealed an overall recurrence rate of 19.5 % (95 % CI = 15.2 to 24.6).

We next examined proband sex and other demographic variables to determine whether they were associated with recurrence rates. Table 1 shows the sample characteristics for each of these variables. Neither proband sex (*X*^*2*^ = 0.59, df = 1, *p* = .44), non-Caucasian status (*X*^*2*^ = 0.36, df = 1, *p* = .55), or paternal education (*X*^*2*^ = 2.09, df = 1, *p* = .15) was significantly associated with ASD recurrence. There was a non-significant trend for maternal education to be associated with recurrence rate (*X*^*2*^ = 3.10, df = 1, *p* = .08). These characteristics did not have significant moderating effects on infant sex or multiplex status in predicting outcome.

To test for younger sibling sex effects, sex was entered as a predictor of dichotomous ASD outcome over and above the random effect for site. The overall effect for sex was significant (*X*^*2*^ = 55.35, df = 1, *p* < .001). The overall percentage of recurrence was 26.7 % for males and 10.3 % for females. The odds ratio of male to female recurrence was 3.18 (95 % CI = 2.31 to 4.39).

**Fig. 1:**
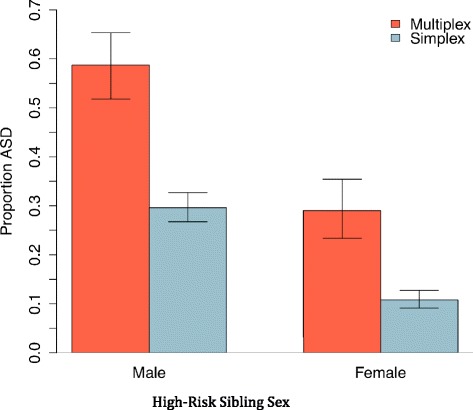
Proportion of ASD outcome by high-risk sibling sex and family multiplex status (±1 SE). *ASD* autism spectrum disorder

The impact of multiplex status was evaluated among the 991 HR infant siblings with data on multiplex status, of whom 77 (7.8 %) were from multiplex families. Adding multiplex status to the model including sex and site revealed a significant main effect for multiplex status (*X*^*2*^ = 20.68, df = 1, *p* < .01), but no interaction between sex and multiplex status (*X*^*2*^ = 0.04, df = 1, *p* = .85). The odds ratio of recurrence in multiplex to simplex families was 3.38 (95 % CI = 2.02 to 5.66). Thus male sex and multiplex status were each independently associated with an approximate 3:1 increase in the odds of ASD recurrence. Figure 1 shows proportions of ASD recurrence in males and females for simplex and multiplex families.

Finally, we examined the interaction between sex of the identified proband and multiplex status among the 403 simplex and 58 multiplex families for whom data were available (see Additional file 1). Despite elevated rates of recurrence for infant siblings from multiplex families in which the identified proband was female, the interaction term was not significant (*X*^*2*^ = 2.71, df = 1, *p* = .10). Given the small sample of female probands in multiplex families (*n* = 7), these analyses should be interpreted with caution,

### Profile analyses

#### Missing data

For both the ADOS and MSEL, missing data were present at all ages for both sexes in all three groups. Levels of missing data tended to be comparable for males and females and to be more common among LR and HR non-ASD than among the ASD group. On the ADOS, for example, 13.9 % of data were missing at 24 months and 14.2 % were missing at 36 months. At 36 months, 14.1 % of male and 14.3 % of female ADOS were missing; likewise, 2.4 % of HR-ASD, 17.2 % of HR-No-ASD, and 14.2 % of LR ASD were missing. These patterns suggest that high-risk status and the emergence of ASD indicators within the HR group are associated with less missing data. As such, estimates of profile scores may be biased slightly toward poorer functioning in the LR non-ASD and HR non-ASD groups, thereby making group comparisons slightly more conservative.

#### MSEL developmental profiles

**Fig. 2:**
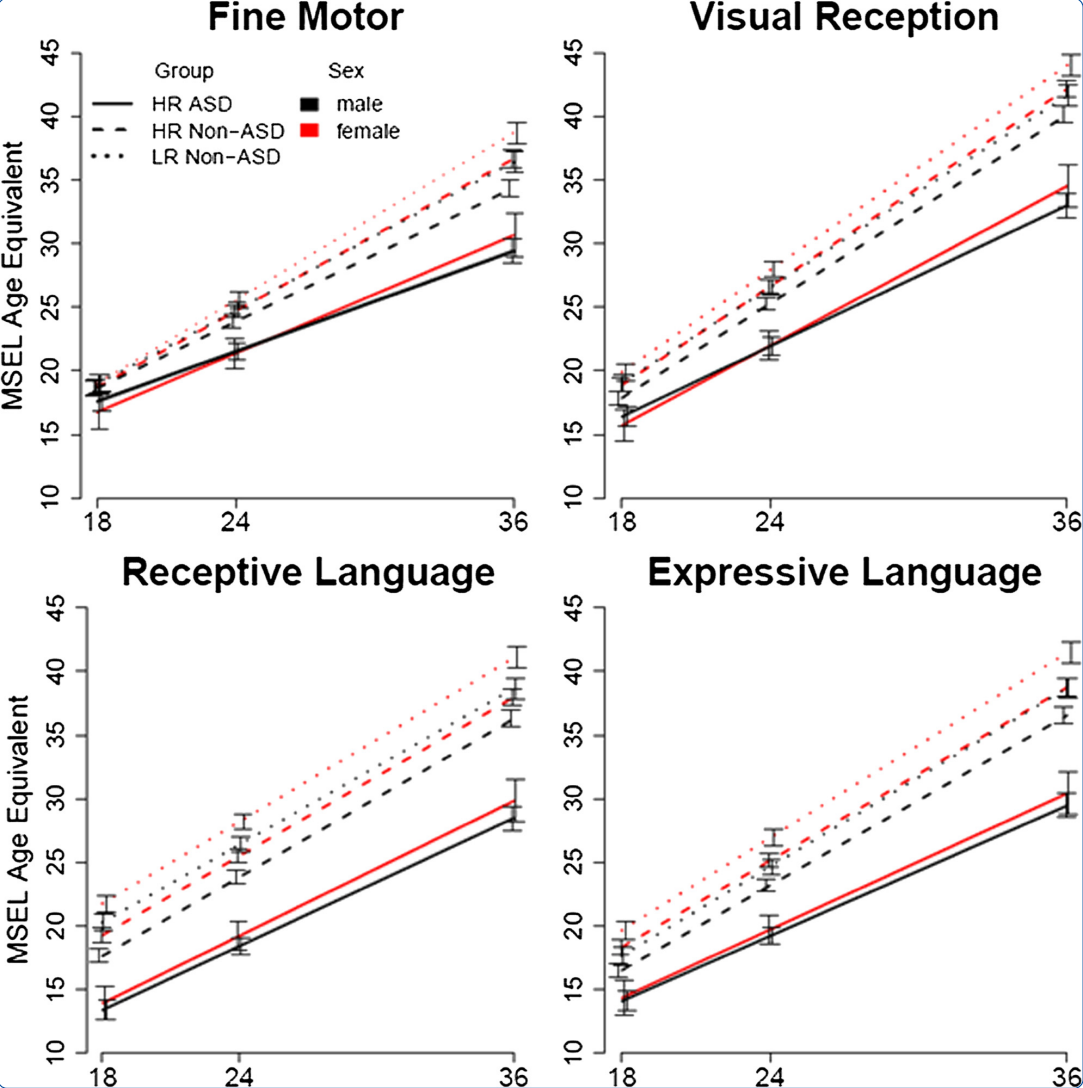
MSEL subscale age-equivalent scores by sex and group (±1 SE). *MSEL* Mullen Scales of Early Learning

Results of the full factorial model of MSEL age-equivalent scores are presented in Table 2. Figure 2 plots estimated marginal means from this model (see Additional file 2). In brief, all main and interaction effects of group, age, and MSEL subscale were significant (*p* < .001). There was a main effect of sex (*p* < .001). However, the sex by group interaction was not significant (*p* = .17), and there were no significant higher-order interactions involving sex and group (all *p* > .62).

**Table 2:** MSEL profile analysis effects

Effect	df	*F* value	*p* value
Main effects			
Subscale	3, 13,141.97	283.41	<.001
Sex	1, 1734.49	77.02	<.001
Group	2, 1751.67	313.07	<.001
Age	1, 1631.21	24,379.20	<.001
Two-way interaction effects			
Subscale × sex	3, 13,158.11	6.57	<.001
Subscale × group	6, 13,179.68	40.49	<.001
Sex × group	2, 1731.83	1.79	.17
Subscale × age	3, 13,144.51	261.42	<.001
Sex × age	1, 1594.41	19.52	<.001
Group × age	2, 1595.04	106.09	<.001
Three-way interaction effects			
Subscale × sex × group	6, 13,135.12	0.48	.82
Subscale × sex × age	3, 13,172.63	7.72	<.001
Subscale × group × age	6, 13,196.43	5.53	<.001
Sex × group × age	2, 1614.78	0.48	.62
Four-way interaction effect			
Subscale × sex × group × age	6, 13,197.46	0.26	.96

The MSEL model contained two significant three-way interactions, each involving a subscale and age. A subscale by sex by age interaction indicated developmental changes in male and female subscale profiles. Simple effects decomposing this three-way interaction are presented in Additional file 3. Slope comparisons between sexes revealed that age equivalent scores increased more rapidly in females than in males for each of the four subscales (all *p* < .05). When age was re-centered at each age to test sex differences within subscales, males were significantly lower than females at each age (all *p* < .001) on all but one subscale (fine motor at 18 months). The female advantage for significant comparisons at each age ranged from 1.06 to 3.3 months on age-equivalent scores; effect sizes ranged from medium to large (*d* range .33 to .54). Examination of simple effects revealed that both males and females showed similar trajectories among different subscales, with visual reception scores rising significantly more rapidly than other subscales, fine motor rising significantly more slowly than other subscales, and both language subscales rising at intermediate rates.

Examination of the simple effects for the three-way interaction (presented in Additional file 4) between outcome group, subscale, and age revealed, as expected, that the HR ASD group had slower growth in all four subscales than each of the other two groups (all *p* < .001). The two non-ASD groups did not differ in their visual reception and receptive language trajectories, but the HR non-ASD group had significantly slower growth in fine motor and expressive language when compared to the LR non-ASD group. Comparing group differences within subscales at each age, the HR ASD group scored significantly below the HR non-ASD group on all subscales at all ages (all *p* < .001), and the HR non-ASD group scored below the LR non-ASD group on all but one subscale (fine motor at 18 months) at all ages (all *p* < .001).

For slope comparisons between subscales within each outcome group, all three groups showed similar patterns of trajectories across the subscales, with the greatest increases in visual reception, the slowest increases in fine motor, and both language subscales showing intermediate growth over time. The HR ASD group, however, did appear to show less differentiation between the trajectories of the language subscales than the two comparison groups.

#### ADOS developmental profiles

**Fig. 3:**
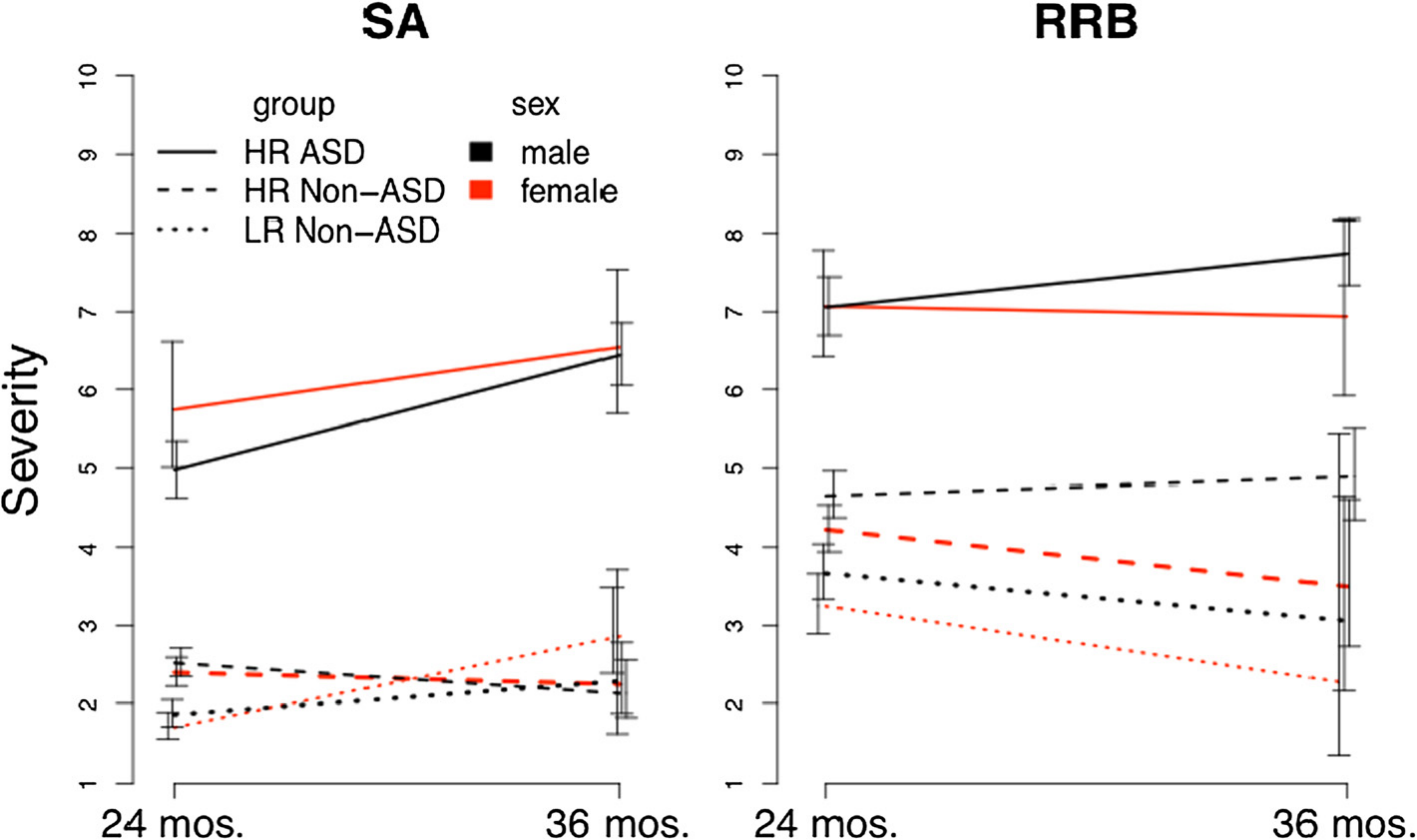
ADOS domain scores for sex and group over age (±1 SE). *SA* social affect, *RRB* restricted and repetitive behavior

Results of the full factorial model for the ADOS domain severity scores are presented in Table 3. Figure 3 displays estimated marginal means (see Additional file 5) for the full factorial model for sex and group in each domain. In brief, the sex by group interaction was not significant (*p* = .27), and there were no significant higher-order interactions involving sex and group (all *p* > .12). As in the MSEL analyses, effects of sex and group were not associated.

**Table 3:** ADOS profile analysis effects

Effect	df	Wald Chi-square	*p* value
Main effects			
Domain	1	58.72	<.001
Sex	1	1.09	.30
Group	2	373.67	<.001
Age	1	0.68	.41
Two-way interaction effects			
Domain × sex	1	4.90	<.05
Domain × group	2	54.74	<.001
Sex × group	2	2.65	.27
Domain × age	1	9.77	<.01
Sex × age	1	0.02	.90
Group × age	2	9.04	<.05
Three-way interaction effects			
Domain × sex × group	2	0.81	.67
Domain × sex × age	1	3.22	.07
Domain × group × age	2	9.47	<.01
Sex × group × age	2	0.29	.86
Four-way interaction effect			
Domain × sex × group × age	2	4.33	.12

There was a significant ADOS domain by sex interaction effect (see Additional file 6 for simple effect comparisons). The comparison of sex within domain revealed that males had significantly higher RRB scores than females, a medium effect size (*d* = .29). SA scores did not differ by sex. Across domains, RRB scores were higher than SA scores for both males and females; this difference was greater for males than for females.

Simple effect comparisons for the significant three-way interaction between group, domain, and age are shown in Additional file 7. The HR ASD group was significantly higher than each of the non-ASD comparison groups at both ages in both RRB and SA by between 2.25 and 4.31 points (all *p* < .001). At 24 months, the HR non-ASD group was significantly higher than the LR non-ASD group in both the RRB and SA domains but, at 36 months, was only higher in the RRB domain. SA severity scores increased significantly for both the ASD and LR non-ASD groups between 24 and 36 months, but not for the HR non-ASD group. No change in RRB over time was observed for any group.

#### Multiplex status and proband sex as predictors in profile analyses

Building upon the profile analysis models, we investigated whether sex of the identified proband and multiplex status influenced MSEL age equivalent scores and ADOS domain scores. For the MSEL subscales, proband sex did not have a significant main effect (*X*^*2*^ = 1.42, df = 1, *p* = .23), nor did it interact with group (*X*^*2*^ = 0.69, df = 1, *p* = .41) or with infant sibling sex (*X*^*2*^ = 0.13, df = 1, *p* = .72). There was a main effect for multiplex status (*X*^*2*^ = 5.33, df = 1, *p* < .05), with infants from multiplex families scoring, on average, 0.84 points (SE = 0.37) lower on MSEL subscales than infants from simplex families. However, multiplex status did not interact with group (*X*^*2*^ = 0.02, df = 1, *p* = .88) or with infant sibling sex (*X*^*2*^ = 1.35, df = 1, *p* = .25).

For the ADOS profile analyses, there was no significant main effect of proband sex (*X*^*2*^ = 0.09, df = 1, *p* = .76), and no interaction with infant sibling sex (*X*^*2*^ = 0.95, df =1, *p* = .33) or group (*X*^*2*^ = 0.19, df = 1, *p* = .66). Similarly, for multiplex status, there was no main effect (*X*^*2*^ = 0.66, df = 1, *p* = .42), and no interaction with infant sibling sex (*X*^*2*^ = 0.18, df = 1, *p* = .67) or with group (*X*^*2*^ = 0.97, df = 1, *p* = .33).

## Discussion

This investigation of 1241 high-risk siblings offers a prospective view of sex differences in ASD risk and the emergence of the ASD phenotype. The odds ratio of ASD recurrence was 3:18 for male versus female high-risk siblings and was not impacted by proband sex. With respect to group differences, as expected, children with ASD performed more poorly on cognitive subscales and exhibited higher levels of ASD-symptom severity than other children. Among high-risk siblings (with and without ASD) and low-risk comparison children, a female rather than a male advantage was evident. Across risk and outcome groups, girls performed better than boys in all dimensions of cognitive functioning assessed and exhibited a lower level of repetitive behavioral severity than boys. As sex differences were neither attenuated nor exaggerated among children with ASD, the results highlight the role of normative sex differences in the development of the autism phenotype. Proband sex was not associated with ASD symptom severity or cognitive functioning, a pattern which does not implicate a female protective effect.

### Sex differences in recurrence

Differences in the prevalence of ASD among males and females are among the most well-documented features of the disorder, but these sex differences vary by sample and ascertainment procedure [3, 8, 33]. Methodological strengths of the current study included prospective tracking of a large sample of 1241 high-risk siblings, 527 of whom were female, recruited by 18 months of age. This is the largest prospectively ascertained sample of infants at elevated risk for ASD due to familial factors to date. Assessment of ASD outcome occurred at a fixed time point, 3 years of age, using both clinical best estimate diagnosis and ADOS criteria. The male rate of ASD recurrence in the high-risk siblings was approximately 1 in 4 (26.7 %) while the female rate was 1 in 10 (10.3 %). The 3.18:1 increased odds of ASD in males in the current sample (95 % CI = 2.31 to 4.39) is similar to estimates from community-based ascertainment of children (2.5:1–2.6:1) [5–7], as well as to a previous report on approximately half of the current high-risk sample [10].

The overall—combined male and female—ASD recurrence rate of 19.5 % yielded an ASD outcome for approximately one in five high-risk siblings. Multiplex status, which characterized 8 % of the current 1241 high-risk siblings, was associated with a threefold increase in ASD risk, underscoring familial risk related to the influence of rare and common genetic variants [34, 35]. The risks associated with a high-risk sibling being male and being from a multiplex status family were independent but cumulative. The dual impact of being a male and being from a multiplex family resulted in an approximately one in two risks of ASD. Although consonant with earlier reports from the sample [10], the number of multiplex cases (*n* = 77) was limited, suggesting that multiplex results should be interpreted with caution.

### Proband sex

Proband sex was not associated with recurrence in the full sample. Although the number of multiplex families with female probands was small, proband sex also did not interact with multiplex status to predict recurrence. The current lack of evidence for a proband sex effect on recurrence mirrors a recent population-based study [8] and reports on clinically diagnosed ASD cases [23, 24]. Although a recent report on twins reported higher recurrence for siblings of female probands, probands (and affected siblings) were identified by thresholding (e.g., greater than 90th percentile) parent-reported autistic traits rather than by diagnosis [22]. Likewise, previous reports that the siblings of female probands present with higher levels of ASD symptoms are based on parent-report [16, 22], while the examiner-administered ADOS in the current investigation did not yield differences in repetitive behaviors or social affect based on sex of proband.

### Sex differences and the early ASD phenotype

In contrast to sex differences in ASD occurrence, relatively little is known about the development of sex differences in the ASD phenotype as it emerges in early childhood. In the current study, sex differences were assayed longitudinally to investigate developmental changes in cognitive functioning and autism symptoms among 1241 high-risk siblings and an additional 583 low-risk children. Challenging accounts of greater female affectedness, there was no evidence that girls exhibited lower levels of cognitive functioning or higher levels of symptom severity than boys. Boys across all groups exhibited slower growth trajectories and lower levels of cognitive performance than girls in fine motor, visual reception, receptive and expressive language functioning. Likewise, children with ASD outcomes, regardless of sex, exhibited slower growth trajectories and lower levels of performance than both high-risk and low-risk children without ASD. However, sex differences and ASD effects were not associated. There was no evidence that boys in the HR ASD group performed disproportionately more poorly than boys in the other two groups. These findings are consistent with reports from large *N* studies of a female advantage in both verbal and nonverbal functioning among low-risk children between 1 and 3 years of age [36–39].

With respect to the ASD symptom severity indices, males exhibited higher levels of repetitive behaviors than females, but there were no sex differences in social affect severity scores. Elevation in restricted and repetitive behaviors in boys is a robust sex difference, evident in both younger [17] and older children with ASD [16]. Children with ASD exhibited elevated levels of both repetitive behaviors and disturbances of social affect with respect to HR siblings without ASD and low-risk children. At 36 months, the high-risk group without ASD outcomes exhibited higher levels of restricted and repetitive behaviors, but not social affect difficulties, than the low-risk group. Specification of this particular area of challenges for non-diagnosed high-risk siblings was not possible in reports of smaller subsets of this sample [26]. Although a meta-analysis of clinical samples suggested that elevated levels of repetitive and stereotyped behaviors in males emerged only after age 6 [40], the current dataset affords a more fine-grained developmental perspective. While social affect severity scores increased for the HR ASD group, these children exhibited stable and elevated levels of restricted and repetitive behaviors between 2 and 3 years. As elevations in restricted and repetitive behaviors were evident at age 2, these behaviors may be helpful in forecasting ASD outcome in both males and females. This pattern of results points to the potential clinical importance of prospective developmental designs in understanding sex differences in the emergence of autism.

No sex differences in social affect symptoms were evident across risk and outcome groups. In low-risk children, a small female advantage in social affect, e.g., more expression of positive emotions with unfamiliar adults, can be detected [41]. Although the ADOS can function as an index of severity, it may have limited sensitivity to detect such subtle effects. Elevations in restricted and repetitive behavior were characteristic of all children with ASD and of boys relative to girls regardless of ASD or risk status. The elevation in restricted and repetitive behavior in male children and male adults with and without ASD is consistent with a male focus on regularity in the behavior of non-social objects and events [19, 42, 43]. The results suggest that male:female ASD differences are not ASD-specific but instead reflect more general sex differences reflected through a prism of autism-linked symptoms [11].

### Limitations

The instruments used to assess sex differences among multiple sites of Baby Sibling Research Consortium were relatively coarse-grained behavioral assays. Neuroimaging and electrophysiological investigations of sex differences in the developing brain, as well as subtler behavioral measures of attention, joint attention, learning, and social interaction may reveal ASD-specific sex differences not documented here. Nevertheless, an advantage in early cognitive functioning and lower levels of restricted and repetitive behaviors were both evident in females. Although there was no overall evidence of a female protective effect, larger samples will be required to address the possibility that female probands in multiplex families (two or more female siblings) confer greater risk for ASD in successive offspring. Recent findings highlight within family diversity in the de novo and rare inherited genetic mutations linked to sibling ASD [44]. One path to greater understanding of sex differences in ASD occurrence and symptomatology will require genetically informed prospective designs which document the potential impact of rare genetic variants on a landscape of continuously distributed enabling and protective factors [21, 34].

## Conclusions

This large prospectively ascertained sample of infants at high risk for ASD due to familial factors revealed a three-to-one male:female odds ratio in ASD recurrence. Children with ASD had lower levels of cognitive functioning and higher symptom severity levels than high-risk children without ASD who, in turn, exhibited lower cognitive functioning and higher ASD symptom severity than low-risk comparison children. Regardless of group membership, males exhibited lower levels of cognitive functioning than females and higher levels of restricted and repetitive behaviors. That is, sex differences were characteristic of the entire longitudinal sample including both high-risk siblings (with and without ASD) and low-risk comparison children. The results suggest that the emergence of ASD symptoms in high-risk siblings—both with and without eventual ASD outcomes—occurs in the context of naturally occurring sex-related variability. There was no evidence, however, that the younger siblings of female probands exhibited greater ASD recurrence or symptoms, casting doubt on a female protective effect among high-risk ASD siblings. For these children, male younger sibling sex remains a robust risk factor for categorical and quantitative impairment.

## Additional files

Additional file 1: Table S1. Recurrence Rates by Proband Sex and Mutliplex Status. Additional file 2: Table S2. MSEL Subscale estimated marginal means. Estimated marginal means for the MSEL subscales for each group by sex by age. Additional file 3: Table S3. MSEL Sex by Subscale by Age simple effects. Slope and intercept comparisons of the MSEL subscale by sex. Additional file 4: Table S4. MSEL Group by Subscale by Age simple effects. Slope and intercept comparisons of the MSEL subscales by group. Additional file 5: Table S5. ADOS Domain estimated marginal means. Estimated marginal means and standard error for ADOS domains (RRB, SA) for each group by sex by age. Additional file 6: Table S6. ADOS Sex by Domain simple effects. ADOS domain comparisons by sex. Additional file 7: Table S7. ADOS Group by Domain by Age simple effects. Comparisons by group, age, and ADOS domain.

## Abbreviations

ADOS: Autism Diagnostic Observation Schedule
ASD: autism spectrum disorder
BSRC: Baby Siblings Research Consortium
CI: confidence interval
HGLM: hierarchical generalized linear model
HR: high-risk group
HR non-ASD: high-risk group without ASD outcomes
HR-ASD: high-risk group with ASD outcomes
IQ: intelligence quotient
LR: low-risk group
MSEL: Mullen Scales of Early Learning
RRB: restricted and repetitive behavior calibrated severity scores from the Autism Diagnostic Observation Schedule
SA: social affect calibrated severity scores from the Autism Diagnostic Observation Schedule
