# Supplementary material for: Early sex differences are not autism-specific: A Baby Siblings Research Consortium (BSRC) study
Source: Mol Autism. 2015 Jun 4;6:32. doi: 10.1186/s13229-015-0027-y (PMC4455973; doi:10.1186/s13229-015-0027-y)
Supplement: Additional file 1: Table S1. — Recurrence Rates by Proband Sex and Mutliplex Status. [file 13229_2015_27_MOESM1_ESM.docx]

**Table S1: Recurrence Rates by Proband Sex and Multiplex Status**

|  |  |  | **Estimate** | | **95% CI** | |
| --- | --- | --- | --- | --- | --- | --- |
| **Multiplex status** | **Proband sex** | **Sample**  **size** | **Probability** | **SE** | **lower bound** | **upper bound** |
| Simplex | Female | 71 | 0.21 | 0.31 | 0.13 | 0.33 |
|  | Male | 332 | 0.21 | 0.18 | 0.15 | 0.27 |
| Multiplex | Female | 7 | 0.72 | 0.88 | 0.31 | 0.94 |
|  | Male | 51 | 0.35 | 0.32 | 0.23 | 0.51 |
