# Supplementary material for: Early sex differences are not autism-specific: A Baby Siblings Research Consortium (BSRC) study
Source: Mol Autism. 2015 Jun 4;6:32. doi: 10.1186/s13229-015-0027-y (PMC4455973; doi:10.1186/s13229-015-0027-y)
Supplement: Additional file 2: Table S2. — MSEL Subscale estimated marginal means. Estimated marginal means for the MSEL subscales for each group by sex by age. [file 13229_2015_27_MOESM2_ESM.docx]

Table S2: MSEL Subscale Estimate Marginal Means.

| **Subscale** | **Group** | **Sex** | **Age** | **Estimate** | **SE** | **Lower Bound** | **Upper Bound** |
| --- | --- | --- | --- | --- | --- | --- | --- |
| Fine Motor | HR  Non-ASD | Female | 18 | 18.72 | 0.29 | 18.16 | 19.29 |
|  |  |  | 24 | 24.70 | 0.26 | 24.19 | 25.22 |
|  |  |  | 36 | 36.67 | 0.34 | 35.99 | 37.34 |
|  |  | Male | 18 | 18.64 | 0.28 | 18.09 | 19.19 |
|  |  |  | 24 | 23.89 | 0.26 | 23.39 | 24.39 |
|  |  |  | 36 | 34.38 | 0.34 | 33.72 | 35.05 |
|  | ASD | Female | 18 | 16.77 | 0.71 | 15.38 | 18.16 |
|  |  |  | 24 | 21.40 | 0.60 | 20.23 | 22.57 |
|  |  |  | 36 | 30.66 | 0.86 | 28.97 | 32.35 |
|  |  | Male | 18 | 17.62 | 0.40 | 16.83 | 18.41 |
|  |  |  | 24 | 21.55 | 0.35 | 20.86 | 22.24 |
|  |  |  | 36 | 29.41 | 0.49 | 28.46 | 30.37 |
|  | LR  Non-ASD | Female | 18 | 19.00 | 0.35 | 18.31 | 19.69 |
|  |  |  | 24 | 25.57 | 0.31 | 24.96 | 26.18 |
|  |  |  | 36 | 38.72 | 0.42 | 37.89 | 39.55 |
|  |  | Male | 18 | 18.95 | 0.35 | 18.26 | 19.65 |
|  |  |  | 24 | 24.77 | 0.31 | 24.16 | 25.38 |
|  |  |  | 36 | 36.40 | 0.42 | 35.59 | 37.22 |
| Expressive Language | HR  Non-ASD | Female | 18 | 18.33 | 0.28 | 17.78 | 18.89 |
|  |  |  | 24 | 25.14 | 0.26 | 24.63 | 25.65 |
|  |  |  | 36 | 38.75 | 0.34 | 38.07 | 39.42 |
|  |  | Male | 18 | 16.51 | 0.28 | 15.97 | 17.06 |
|  |  |  | 24 | 23.19 | 0.26 | 22.69 | 23.69 |
|  |  |  | 36 | 36.55 | 0.34 | 35.88 | 37.21 |
|  | ASD | Female | 18 | 14.36 | 0.70 | 12.99 | 15.72 |
|  |  |  | 24 | 19.71 | 0.59 | 18.55 | 20.86 |
|  |  |  | 36 | 30.41 | 0.86 | 28.72 | 32.10 |
|  |  | Male | 18 | 14.09 | 0.40 | 13.31 | 14.87 |
|  |  |  | 24 | 19.21 | 0.35 | 18.53 | 19.90 |
|  |  |  | 36 | 29.45 | 0.49 | 28.50 | 30.40 |
|  | LR  Non-ASD | Female | 18 | 19.65 | 0.35 | 18.97 | 20.34 |
|  |  |  | 24 | 26.93 | 0.31 | 26.32 | 27.53 |
|  |  |  | 36 | 41.47 | 0.42 | 40.64 | 42.30 |
|  |  | Male | 18 | 17.65 | 0.35 | 16.96 | 18.33 |
|  |  |  | 24 | 24.66 | 0.31 | 24.06 | 25.26 |
|  |  |  | 36 | 38.68 | 0.42 | 37.87 | 39.50 |
| Receptive Language | HR  Non-ASD | Female | 18 | 19.27 | 0.28 | 18.73 | 19.82 |
|  |  |  | 24 | 25.50 | 0.26 | 25.00 | 26.00 |
|  |  |  | 36 | 37.95 | 0.34 | 37.28 | 38.63 |
|  |  | Male | 18 | 17.63 | 0.27 | 17.10 | 18.17 |
|  |  |  | 24 | 23.85 | 0.25 | 23.35 | 24.34 |
|  |  |  | 36 | 36.27 | 0.34 | 35.61 | 36.93 |
|  | ASD | Female | 18 | 13.92 | 0.66 | 12.63 | 15.22 |
|  |  |  | 24 | 19.22 | 0.57 | 18.10 | 20.35 |
|  |  |  | 36 | 29.82 | 0.86 | 28.13 | 31.51 |
|  |  | Male | 18 | 13.39 | 0.38 | 12.64 | 14.14 |
|  |  |  | 24 | 18.42 | 0.34 | 17.74 | 19.09 |
|  |  |  | 36 | 28.47 | 0.49 | 27.51 | 29.43 |
|  | LR  Non-ASD | Female | 18 | 21.76 | 0.34 | 21.09 | 22.42 |
|  |  |  | 24 | 28.19 | 0.30 | 27.60 | 28.79 |
|  |  |  | 36 | 41.06 | 0.42 | 40.23 | 41.89 |
|  |  | Male | 18 | 20.27 | 0.33 | 19.61 | 20.92 |
|  |  |  | 24 | 26.39 | 0.30 | 25.80 | 26.98 |
|  |  |  | 36 | 38.64 | 0.42 | 37.82 | 39.46 |
| Visual Reception | HR  Non-ASD | Female | 18 | 18.89 | 0.28 | 18.34 | 19.43 |
|  |  |  | 24 | 26.65 | 0.26 | 26.15 | 27.16 |
|  |  |  | 36 | 42.18 | 0.34 | 41.50 | 42.85 |
|  |  | Male | 18 | 17.88 | 0.27 | 17.34 | 18.41 |
|  |  |  | 24 | 25.30 | 0.25 | 24.80 | 25.79 |
|  |  |  | 36 | 40.14 | 0.34 | 39.48 | 40.80 |
|  | ASD | Female | 18 | 15.72 | 0.66 | 14.43 | 17.02 |
|  |  |  | 24 | 21.99 | 0.57 | 20.87 | 23.11 |
|  |  |  | 36 | 34.53 | 0.86 | 32.85 | 36.22 |
|  |  | Male | 18 | 16.42 | 0.38 | 15.67 | 17.16 |
|  |  |  | 24 | 21.95 | 0.34 | 21.28 | 22.62 |
|  |  |  | 36 | 33.01 | 0.49 | 32.06 | 33.97 |
|  | LR  Non-ASD | Female | 18 | 19.91 | 0.34 | 19.25 | 20.57 |
|  |  |  | 24 | 27.95 | 0.30 | 27.35 | 28.54 |
|  |  |  | 36 | 44.02 | 0.42 | 43.19 | 44.85 |
|  |  | Male | 18 | 19.05 | 0.33 | 18.39 | 19.70 |
|  |  |  | 24 | 26.57 | 0.30 | 25.98 | 27.16 |
|  |  |  | 36 | 41.61 | 0.42 | 40.80 | 42.43 |
