# Supplementary material for: Early sex differences are not autism-specific: A Baby Siblings Research Consortium (BSRC) study
Source: Mol Autism. 2015 Jun 4;6:32. doi: 10.1186/s13229-015-0027-y (PMC4455973; doi:10.1186/s13229-015-0027-y)
Supplement: Additional file 3: Table S3. — MSEL Sex by Subscale by Age simple effects. Slope and intercept comparisons of the MSEL subscale by sex. [file 13229_2015_27_MOESM3_ESM.docx]

Table S3: MSEL Sex by Subscale by Age simple effects.

| **Slope comparisons between Sex within Subscales** | | | | | |  |  | |  | | |  | | |  | |  | |
| --- | --- | --- | --- | --- | --- | --- | --- | --- | --- | --- | --- | --- | --- | --- | --- | --- | --- | --- |
|  |  | **Subscale** | | **Comparison** | | **Difference** | **SE** | **df** | | | | | **t-value** | | | **p-value^a^** | | **Effect (d)** |
|  |  | | Fine Motor | | Male vs Female | -0.15 | 0.0204 | | 5700.77 | | | -7.60 | | | p < .0008* | | 0.31 | |
|  |  | | Visual Reception | | Male vs Female | -0.11 | 0.0198 | | 5194.90 | | | -5.69 | | | p < .0008* | | 0.17 | |
|  |  | | Receptive Language | | Male vs Female | -0.05 | 0.0198 | | 5225.85 | | | -2.41 | | | p = 0.0161 | | 0.09 | |
|  |  | | Expressive Language | | Male vs Female | -0.07 | 0.0202 | | 5537.60 | | | -3.28 | | | p = 0.0011 | | 0.12 | |
| **Slope comparisons between Subscales within Sex** | | | | | |  |  | |  | | |  | | |  | |  | |
|  |  | **Sex** | | **Comparison** | | **Difference** | **SE** | | | **df** | **t-value** | | | **p-value** | | | | **Effect (d)** |
|  |  | | Female | | EL^b^ vs FM | 0.13 | 0.0173 | | 13145.38 | | | 7.50 | | | p < .0008* | | 0.22 | |
|  |  | |  |  | RL vs FM | 0.03 | 0.0171 | | 13225.14 | | | 1.50 | | | p = 0.1340 | | 0.05 | |
|  |  | |  |  | VR vs FM | 0.28 | 0.0171 | | 13219.09 | | | 16.25 | | | p < .0008* | | 0.47 | |
|  |  | |  |  | EL vs VR | -0.15 | 0.0169 | | 13180.59 | | | -8.73 | | | p < .0008* | | 0.25 | |
|  |  | |  |  | RL vs VR | -0.25 | 0.0167 | | 13099.02 | | | -15.05 | | | p < .0008* | | 0.42 | |
|  |  | |  |  | EL vs RL | 0.10 | 0.0170 | | 13180.60 | | | 6.12 | | | p < .0008* | | 0.17 | |
|  |  | | Male | | EL vs FM | 0.22 | 0.0155 | | 13144.73 | | | 14.03 | | | p < .0008* | | 0.38 | |
|  |  | |  |  | RL vs FM | 0.13 | 0.0154 | | 13236.68 | | | 8.63 | | | p < .0008* | | 0.23 | |
|  |  | |  |  | VR vs FM | 0.32 | 0.0153 | | 13231.49 | | | 20.84 | | | p < .0008* | | 0.56 | |
|  |  | |  |  | EL vs VR | -0.10 | 0.0152 | | 13215.18 | | | -6.67 | | | p < .0008* | | 0.17 | |
|  |  | |  |  | RL vs VR | -0.19 | 0.0150 | | 13120.05 | | | -12.46 | | | p < .0008* | | 0.33 | |
|  |  | |  |  | EL vs RL | 0.09 | 0.0153 | | 13203.10 | | | 5.59 | | | p < .0008* | | 0.16 | |
| **Intercept comparisons between Sex within Subscales** | | | | | |  |  | |  | | |  | | |  | |  | |
| **Subscale** | | **Age Point** | | **Comparison** | | **Difference** | **SE** | | | **df** | **t-value** | | | **p-value** | | | | **Effect (d)** |
|  | Fine Motor | | 18 | | Male vs Female | -0.13 | 0.2451 | | 7141.79 | | | -0.54 | | | p = 0.5864 | | 0.06 | |
|  |  |  | 24 | | Male vs Female | -1.06 | 0.2182 | | 3886.85 | | | -4.87 | | | p < .0008* | | 0.33 | |
|  |  |  | 36 | | Male vs Female | -2.92 | 0.3352 | | 2836.69 | | | -8.71 | | | p < .0008* | | 0.46 | |
|  | Visual Reception | | 18 | | Male vs Female | -1.07 | 0.2322 | | 6217.00 | | | -4.60 | | | p < .0008* | | 0.36 | |
|  |  |  | 24 | | Male vs Female | -1.74 | 0.2123 | | 3533.14 | | | -8.21 | | | p < .0008* | | 0.35 | |
|  |  |  | 36 | | Male vs Female | -3.09 | 0.3347 | | 2821.84 | | | -9.24 | | | p < .0008* | | 0.35 | |
|  | Receptive Language | | 18 | | Male vs Female | -2.17 | 0.2325 | | 6238.10 | | | -9.33 | | | p < .0008* | | 0.41 | |
|  |  |  | 24 | | Male vs Female | -2.46 | 0.2125 | | 3543.33 | | | -11.56 | | | p < .0008* | | 0.41 | |
|  |  |  | 36 | | Male vs Female | -3.03 | 0.3350 | | 2831.20 | | | -9.04 | | | p < .0008* | | 0.37 | |
|  | Expressive Language | | 18 | | Male vs Female | -2.13 | 0.2406 | | 6812.04 | | | -8.85 | | | p < .0008* | | 0.54 | |
|  |  |  | 24 | | Male vs Female | -2.53 | 0.2160 | | 3752.02 | | | -11.69 | | | p < .0008* | | 0.43 | |
|  |  |  | 36 | | Male vs Female | -3.32 | 0.3350 | | 2829.01 | | | -9.91 | | | p < .0008* | | 0.44 | |
| **Intercept comparisons between Subscales within Sex** | | | | | |  |  | | |  | | |  |  | | | |  |
| **Sex** | | **Age Point** | | **Comparison** | | **Difference** | **SE** | | | **df** | **t-value** | | | **p-value** | | | | **Effect (d)** |
|  | Female | | 18 | | EL vs FM | -0.18 | 0.2220 | | 13180.03 | | | -0.81 | | | p = 0.4200 | | 0.05 | |
|  |  | |  | | RL vs FM | 1.05 | 0.2184 | | 13309.54 | | | 4.79 | | | p < .0008* | | 0.27 | |
|  |  | |  | | VR vs FM | 0.33 | 0.2183 | | 13304.44 | | | 1.49 | | | p = 0.1356 | | 0.09 | |
|  |  | |  | | EL vs VR | -0.50 | 0.2154 | | 13240.82 | | | -2.34 | | | p = 0.0191 | | 0.13 | |
|  |  | |  | | RL vs VR | 0.72 | 0.2107 | | 13101.89 | | | 3.42 | | | p < .0008* | | 0.19 | |
|  |  | |  | | EL vs RL | -1.22 | 0.2155 | | 13238.40 | | | -5.68 | | | p < .0008* | | 0.31 | |
|  |  | | 24 | | EL vs FM | 0.60 | 0.1533 | | 13169.55 | | | 3.90 | | | p < .0008* | | 0.12 | |
|  |  | |  | | RL vs FM | 1.20 | 0.1512 | | 13278.25 | | | 7.93 | | | p < .0008* | | 0.24 | |
|  |  | |  | | VR vs FM | 1.99 | 0.1511 | | 13273.92 | | | 13.17 | | | p < .0008* | | 0.40 | |
|  |  | |  | | EL vs VR | -1.39 | 0.1493 | | 13217.87 | | | -9.33 | | | p < .0008* | | 0.28 | |
|  |  | |  | | RL vs VR | -0.79 | 0.1467 | | 13100.46 | | | -5.39 | | | p < .0008* | | 0.16 | |
|  |  | |  | | EL vs RL | -0.60 | 0.1494 | | 13214.76 | | | -4.03 | | | p < .0008* | | 0.12 | |
|  |  | | 36 | | EL vs FM | 2.15 | 0.1904 | | 13097.48 | | | 11.29 | | | p < .0008* | | 0.29 | |
|  |  | |  | | RL vs FM | 1.51 | 0.1906 | | 13099.40 | | | 7.91 | | | p < .0008* | | 0.21 | |
|  |  | |  | | VR vs FM | 5.32 | 0.1903 | | 13094.08 | | | 27.95 | | | p < .0008* | | 0.72 | |
|  |  | |  | | EL vs VR | -3.17 | 0.1901 | | 13092.71 | | | -16.67 | | | p < .0008* | | 0.43 | |
|  |  | |  | | RL vs VR | -3.81 | 0.1902 | | 13094.50 | | | -20.05 | | | p < .0008* | | 0.52 | |
|  |  | |  | | EL vs RL | 0.64 | 0.1903 | | 13093.34 | | | 3.39 | | | p < .0008* | | 0.09 | |
|  | Male | | 18 | | EL vs FM | -2.17 | 0.2010 | | 13172.99 | | | -10.82 | | | p < .0008* | | 0.56 | |
|  |  | |  | | RL vs FM | -0.99 | 0.1971 | | 13319.88 | | | -5.03 | | | p < .0008* | | 0.26 | |
|  |  | |  | | VR vs FM | -0.61 | 0.1969 | | 13315.97 | | | -3.09 | | | p = 0.0020 | | 0.16 | |
|  |  | |  | | EL vs VR | -1.57 | 0.1945 | | 13286.05 | | | -8.05 | | | p < .0008* | | 0.40 | |
|  |  | |  | | RL vs VR | -0.38 | 0.1893 | | 13127.69 | | | -2.02 | | | p = 0.0430 | | 0.10 | |
|  |  | |  | | EL vs RL | -1.18 | 0.1947 | | 13266.78 | | | -6.08 | | | p < .0008* | | 0.30 | |
|  |  | | 24 | | EL vs FM | -0.87 | 0.1391 | | 13162.47 | | | -6.22 | | | p < .0008* | | 0.17 | |
|  |  | |  | | RL vs FM | -0.20 | 0.1369 | | 13288.99 | | | -1.43 | | | p = 0.1539 | | 0.04 | |
|  |  | |  | | VR vs FM | 1.31 | 0.1367 | | 13284.81 | | | 9.58 | | | p < .0008* | | 0.25 | |
|  |  | |  | | EL vs VR | -2.18 | 0.1352 | | 13255.79 | | | -16.09 | | | p < .0008* | | 0.42 | |
|  |  | |  | | RL vs VR | -1.51 | 0.1322 | | 13121.19 | | | -11.39 | | | p < .0008* | | 0.29 | |
|  |  | |  | | EL vs RL | -0.67 | 0.1353 | | 13239.93 | | | -4.96 | | | p < .0008* | | 0.13 | |
|  |  | | 36 | | EL vs FM | 1.75 | 0.1717 | | 13101.68 | | | 10.20 | | | p < .0008* | | 0.21 | |
|  |  | |  | | RL vs FM | 1.40 | 0.1718 | | 13111.68 | | | 8.13 | | | p < .0008* | | 0.17 | |
|  |  | |  | | VR vs FM | 5.15 | 0.1714 | | 13104.93 | | | 30.02 | | | p < .0008* | | 0.62 | |
|  |  | |  | | EL vs VR | -3.40 | 0.1713 | | 13103.75 | | | -19.82 | | | p < .0008* | | 0.41 | |
|  |  | |  | | RL vs VR | -3.75 | 0.1714 | | 13103.26 | | | -21.88 | | | p < .0008* | | 0.45 | |
|  |  | |  | | EL vs RL | 0.35 | 0.1716 | | 13103.80 | | | 2.07 | | | p = 0.0389 | | 0.04 | |

^a^Significance using Bonferroni correction for alpha (.05/64).

^b^EL=Expressive Language; RL=Receptive Language; FM=Fine Motor; VR=Visual Reception.
