# Supplementary material for: Early sex differences are not autism-specific: A Baby Siblings Research Consortium (BSRC) study
Source: Mol Autism. 2015 Jun 4;6:32. doi: 10.1186/s13229-015-0027-y (PMC4455973; doi:10.1186/s13229-015-0027-y)
Supplement: Additional file 4: Table S4. — MSEL Group by Subscale by Age simple effects. Slope and intercept comparisons of the MSEL subscales by group. [file 13229_2015_27_MOESM4_ESM.docx]

Table S4: MSEL Group by Subscale by Age simple effects.

| **Slope comparisons between Group within Subscale** | | | | | |  |  |  |  |  |  |
| --- | --- | --- | --- | --- | --- | --- | --- | --- | --- | --- | --- |
|  |  | **Subscale** | | **Comparison** | | **Difference** | **SE** | **df** | **t-value** | **p-value ^a^** | **Effect (d)** |
|  |  | | Fine Motor | | LR Non-ASD vs ASD | 0.35 | 0.0317 | 6310.23 | 11.05 | p < .0004* | 0.72 |
|  |  | |  |  | HR Non-ASD vs ASD | 0.25 | 0.0295 | 6214.32 | 8.58 | p < .0004* | 0.52 |
|  |  | |  |  | LR Non-ASD vs HR Non-ASD | 0.10 | 0.0222 | 6187.09 | 4.40 | p < .0004* | 0.21 |
|  |  | | Visual Reception | | LR Non-ASD vs ASD | 0.35 | 0.0305 | 5594.87 | 11.36 | p < .0004* | 0.54 |
|  |  | |  |  | HR Non-ASD vs ASD | 0.32 | 0.0284 | 5554.41 | 11.10 | p < .0004* | 0.49 |
|  |  | |  |  | LR Non-ASD vs HR Non-ASD | 0.03 | 0.0215 | 5579.97 | 1.45 | p = 0.1476 | 0.05 |
|  |  | | Receptive Language | | LR Non-ASD vs ASD | 0.20 | 0.0307 | 5702.13 | 6.46 | p < .0004* | 0.34 |
|  |  | |  |  | HR Non-ASD vs ASD | 0.19 | 0.0286 | 5663.83 | 6.61 | p < .0004* | 0.32 |
|  |  | |  |  | LR Non-ASD vs HR Non-ASD | 0.01 | 0.0215 | 5609.88 | 0.44 | p = 0.6606 | 0.02 |
|  |  | | Expressive Language | | LR Non-ASD vs ASD | 0.33 | 0.0314 | 6118.58 | 10.42 | p < .0004* | 0.55 |
|  |  | |  |  | HR Non-ASD vs ASD | 0.26 | 0.0292 | 6033.62 | 8.96 | p < .0004* | 0.44 |
|  |  | |  |  | LR Non-ASD vs HR Non-ASD | 0.07 | 0.0220 | 6002.51 | 2.99 | p = 0.0028 | 0.12 |
| **Slope comparisons between Subscale within Group** | | | | | |  |  |  |  |  |  |
|  |  | **Group** | | **Comparison** | | **Difference** | **SE** | **df** | **t-value** | **p-value** | **Effect (d)** |
|  |  | | ASD | | EL^b^ vs FM | 0.18 | 0.0308 | 13136.05 | 5.89 | p < .0004* | 0.32 |
|  |  | |  |  | RL vs FM | 0.17 | 0.0304 | 13306.47 | 5.49 | p < .0004* | 0.30 |
|  |  | |  |  | VR vs FM | 0.27 | 0.0302 | 13308.15 | 8.88 | p < .0004* | 0.48 |
|  |  | |  |  | EL vs VR | -0.09 | 0.0300 | 13276.01 | -2.90 | p = 0.0037 | 0.16 |
|  |  | |  |  | RL vs VR | -0.10 | 0.0295 | 13143.54 | -3.43 | p = 0.0006 | 0.18 |
|  |  | |  |  | EL vs RL | 0.01 | 0.0302 | 13256.85 | 0.47 | p = 0.6357 | 0.02 |
|  |  | | HR Non-ASD | | EL vs FM | 0.19 | 0.0154 | 13130.98 | 12.33 | p < .0004* | 0.33 |
|  |  | |  |  | RL vs FM | 0.10 | 0.0153 | 13204.20 | 6.75 | p < .0004* | 0.18 |
|  |  | |  |  | VR vs FM | 0.33 | 0.0153 | 13195.34 | 21.66 | p < .0004* | 0.58 |
|  |  | |  |  | EL vs VR | -0.14 | 0.0152 | 13167.57 | -9.28 | p < .0004* | 0.25 |
|  |  | |  |  | RL vs VR | -0.23 | 0.0150 | 13093.81 | -15.18 | p < .0004* | 0.40 |
|  |  | |  |  | EL vs RL | 0.09 | 0.0152 | 13166.04 | 5.72 | p < .0004* | 0.16 |
|  |  | | LR Non-ASD | | EL vs FM | 0.16 | 0.0207 | 13147.66 | 7.65 | p < .0004* | 0.27 |
|  |  | |  |  | RL vs FM | 0.01 | 0.0204 | 13242.64 | 0.73 | p = 0.4667 | 0.02 |
|  |  | |  |  | VR vs FM | 0.26 | 0.0203 | 13237.51 | 12.99 | p < .0004* | 0.44 |
|  |  | |  |  | EL vs VR | -0.11 | 0.0202 | 13216.45 | -5.27 | p < .0004* | 0.19 |
|  |  | |  |  | RL vs VR | -0.25 | 0.0198 | 13097.98 | -12.58 | p < .0004* | 0.43 |
|  |  | |  |  | EL vs RL | 0.14 | 0.0202 | 13208.62 | 7.08 | p < .0004* | 0.24 |
| **Intercept comparisons between Group within Subscale** | | | | | |  |  |  |  |  |  |
| **Subscale** | | **Age Point** | | **Comparison** | | **Difference** | **SE** | **df** | **t-value** | **p-value** | **Effect (d)** |
|  | Fine Motor | | 18 | | LR Non-ASD vs ASD | 1.53 | 0.3917 | 7694.09 | 3.91 | p < .0004* | 0.74 |
|  |  |  |  | | HR Non-ASD vs ASD | 1.25 | 0.3594 | 7731.13 | 3.48 | p = 0.0005 | 0.61 |
|  |  |  |  | | LR Non-ASD vs HR Non-ASD | 0.28 | 0.2728 | 7297.92 | 1.03 | p = 0.3047 | 0.14 |
|  |  | | 24 | | LR Non-ASD vs ASD | 3.63 | 0.3324 | 4504.62 | 10.93 | p < .0004* | 1.13 |
|  |  | |  | | HR Non-ASD vs ASD | 2.77 | 0.3054 | 4493.33 | 9.06 | p < .0004* | 0.86 |
|  |  | |  | | LR Non-ASD vs HR Non-ASD | 0.87 | 0.2333 | 4324.66 | 3.71 | p < .0004* | 0.27 |
|  |  | | 36 | | LR Non-ASD vs ASD | 7.84 | 0.4920 | 3011.57 | 15.94 | p < .0004* | 1.23 |
|  |  | |  | | HR Non-ASD vs ASD | 5.80 | 0.4572 | 2985.40 | 12.69 | p < .0004* | 0.91 |
|  |  | |  | | LR Non-ASD vs HR Non-ASD | 2.04 | 0.3482 | 3097.66 | 5.86 | p < .0004* | 0.32 |
|  | Visual Reception | | 18 | | LR Non-ASD vs ASD | 3.20 | 0.3641 | 6472.96 | 8.78 | p < .0004* | 1.09 |
|  |  |  |  | | HR Non-ASD vs ASD | 2.10 | 0.3351 | 6568.54 | 6.27 | p < .0004* | 0.71 |
|  |  |  |  | | LR Non-ASD vs HR Non-ASD | 1.10 | 0.2569 | 6291.49 | 4.27 | p < .0004* | 0.37 |
|  |  | | 24 | | LR Non-ASD vs ASD | 5.28 | 0.3192 | 3937.92 | 16.53 | p < .0004* | 1.07 |
|  |  | |  | | HR Non-ASD vs ASD | 3.99 | 0.2938 | 3950.86 | 13.59 | p < .0004* | 0.81 |
|  |  | |  | | LR Non-ASD vs HR Non-ASD | 1.28 | 0.2258 | 3876.08 | 5.69 | p < .0004* | 0.26 |
|  |  | | 36 | | LR Non-ASD vs ASD | 9.43 | 0.4915 | 3000.48 | 19.19 | p < .0004* | 1.07 |
|  |  | |  | | HR Non-ASD vs ASD | 7.78 | 0.4568 | 2974.14 | 17.03 | p < .0004* | 0.88 |
|  |  | |  | | LR Non-ASD vs HR Non-ASD | 1.65 | 0.2357 | 3088.40 | 7.00 | p < .0004* | 0.19 |
|  | Receptive Language | | 18 | | LR Non-ASD vs ASD | 7.48 | 0.3669 | 6582.74 | 20.40 | p < .0004* | 1.40 |
|  |  |  |  | | HR Non-ASD vs ASD | 4.92 | 0.3378 | 6684.61 | 14.56 | p < .0004* | 0.92 |
|  |  |  |  | | LR Non-ASD vs HR Non-ASD | 2.57 | 0.2572 | 6306.93 | 9.98 | p < .0004* | 0.48 |
|  |  | | 24 | | LR Non-ASD vs ASD | 8.68 | 0.3206 | 3991.12 | 27.06 | p < .0004* | 1.46 |
|  |  | |  | | HR Non-ASD vs ASD | 6.05 | 0.2951 | 4008.26 | 20.50 | p < .0004* | 1.02 |
|  |  | |  | | LR Non-ASD vs HR Non-ASD | 2.62 | 0.2259 | 3883.49 | 11.61 | p < .0004* | 0.44 |
|  |  | | 36 | | LR Non-ASD vs ASD | 11.06 | 0.4926 | 3023.57 | 22.45 | p < .0004* | 1.35 |
|  |  | |  | | HR Non-ASD vs ASD | 8.32 | 0.4578 | 2998.30 | 18.17 | p < .0004* | 1.02 |
|  |  | |  | | LR Non-ASD vs HR Non-ASD | 2.74 | 0.3479 | 3088.78 | 7.87 | p < .0004* | 0.33 |
|  | Expressive Language | | 18 | | LR Non-ASD vs ASD | 4.49 | 0.3838 | 7373.36 | 11.70 | p < .0004* | 1.14 |
|  |  |  |  | | HR Non-ASD vs ASD | 3.24 | 0.3525 | 7432.59 | 9.20 | p < .0004* | 0.82 |
|  |  |  |  | | LR Non-ASD vs HR Non-ASD | 1.25 | 0.2671 | 6940.39 | 4.67 | p < .0004* | 0.32 |
|  |  | | 24 | | LR Non-ASD vs ASD | 6.45 | 0.3283 | 4327.21 | 19.66 | p < .0004* | 1.10 |
|  |  | |  | | HR Non-ASD vs ASD | 4.81 | 0.3018 | 4325.54 | 15.95 | p < .0004* | 0.82 |
|  |  | |  | | LR Non-ASD vs HR Non-ASD | 1.64 | 0.2305 | 4153.49 | 7.12 | p < .0004* | 0.28 |
|  |  | | 36 | | LR Non-ASD vs ASD | 10.38 | 0.4917 | 3003.71 | 21.11 | p < .0004* | 1.24 |
|  |  | |  | | HR Non-ASD vs ASD | 7.95 | 0.4567 | 2973.45 | 17.41 | p < .0004* | 0.95 |
|  |  | |  | | LR Non-ASD vs HR Non-ASD | 2.43 | 0.3480 | 3092.57 | 6.98 | p < .0004* | 0.29 |
| **Intercept comparisons between Subscale within Group** | | | | | |  |  |  |  |  |  |
| **Group** | | **Age Point** | | **Comparison** | | **Difference** | **SE** | **df** | **t-value** | **p-value** | **Effect (d)** |
|  | ASD | | 18 | | EL vs FM | -3.29 | 0.4035 | 13171.28 | -8.14 | p < .0004* | 0.75 |
|  |  | |  | | RL vs FM | -3.93 | 0.3942 | 13413.31 | -9.96 | p < .0004* | 0.90 |
|  |  | |  | | VR vs FM | -1.17 | 0.3920 | 13419.81 | -2.97 | p = 0.0029 | 0.27 |
|  |  | |  | | EL vs VR | -2.12 | 0.3869 | 13369.46 | -5.48 | p < .0004* | 0.48 |
|  |  | |  | | RL vs VR | -2.76 | 0.3748 | 13152.01 | -7.37 | p < .0004* | 0.63 |
|  |  | |  | | EL vs RL | 0.64 | 0.3891 | 13343.04 | 1.65 | p = 0.0996 | 0.15 |
|  |  | | 24 | | EL vs FM | -2.20 | 0.2772 | 13165.11 | -7.93 | p < .0004* | 0.38 |
|  |  | |  | | RL vs FM | -2.92 | 0.2718 | 13369.40 | -10.76 | p < .0004* | 0.49 |
|  |  | |  | | VR vs FM | 0.44 | 0.2705 | 13370.94 | 1.64 | p = 0.1011 | 0.08 |
|  |  | |  | | EL vs VR | -2.64 | 0.2672 | 13325.37 | -9.89 | p < .0004* | 0.45 |
|  |  | |  | | RL vs VR | -3.37 | 0.2602 | 13135.88 | -12.94 | p < .0004* | 0.57 |
|  |  | |  | | EL vs RL | 0.73 | 0.2685 | 13305.56 | 2.71 | p = 0.0068 | 0.12 |
|  |  | | 36 | | EL vs FM | -0.02 | 0.3305 | 13091.63 | -0.07 | p = 0.9458 | 0.002 |
|  |  | |  | | RL vs FM | -0.92 | 0.3324 | 13122.22 | -2.77 | p = 0.0056 | 0.10 |
|  |  | |  | | VR vs FM | 3.66 | 0.3309 | 13109.98 | 11.07 | p < .0004* | 0.36 |
|  |  | |  | | EL vs VR | -3.69 | 0.3304 | 13108.89 | -11.16 | p < .0004* | 0.36 |
|  |  | |  | | RL vs VR | -4.58 | 0.3319 | 13107.33 | -13.81 | p < .0004* | 0.45 |
|  |  | |  | | EL vs RL | 0.90 | 0.3317 | 13109.40 | 2.71 | p = 0.0068 | 0.09 |
|  | HR  Non-ASD | | 18 | | EL vs FM | -1.29 | 0.1958 | 13159.80 | -6.60 | p < .0004* | 0.34 |
|  |  |  |  | | RL vs FM | -0.26 | 0.1929 | 13279.56 | -1.35 | p = 0.1774 | 0.07 |
|  |  |  |  | | VR vs FM | -0.32 | 0.1930 | 13271.21 | -1.64 | p = 0.1004 | 0.09 |
|  |  |  |  | | EL vs VR | -0.98 | 0.1908 | 13227.49 | -5.11 | p < .0004* | 0.26 |
|  |  |  |  | | RL vs VR | 0.06 | 0.1871 | 13097.13 | 0.30 | p = 0.7612 | 0.02 |
|  |  |  |  | | EL vs RL | -1.03 | 0.1907 | 13216.41 | -5.41 | p < .0004* | 0.27 |
|  |  | | 24 | | EL vs FM | -0.15 | 0.1358 | 13149.25 | -1.13 | p = 0.2603 | 0.03 |
|  |  | |  | | RL vs FM | 0.36 | 0.1342 | 13249.25 | 2.67 | p = 0.0075 | 0.08 |
|  |  | |  | | VR vs FM | 1.67 | 0.1342 | 13243.61 | 12.43 | p < .0004* | 0.36 |
|  |  | |  | | EL vs VR | -1.82 | 0.1328 | 13205.84 | -13.71 | p < .0004* | 0.39 |
|  |  | |  | | RL vs VR | -1.31 | 0.1307 | 13096.41 | -10.02 | p < .0004* | 0.28 |
|  |  | |  | | EL vs RL | -0.51 | 0.1328 | 13193.52 | -3.85 | p < .0004* | 0.11 |
|  |  | | 36 | | EL vs FM | 2.13 | 0.1731 | 13090.44 | 12.29 | p < .0004* | 0.31 |
|  |  | |  | | RL vs FM | 1.60 | 0.1731 | 13094.58 | 9.22 | p < .0004* | 0.23 |
|  |  | |  | | VR vs FM | 5.64 | 0.1729 | 13090.20 | 32.60 | p < .0004* | 0.81 |
|  |  | |  | | EL vs VR | -3.51 | 0.1727 | 13087.02 | -20.33 | p < .0004* | 0.50 |
|  |  | |  | | RL vs VR | -4.04 | 0.1727 | 13090.42 | -23.41 | p < .0004* | 0.58 |
|  |  | |  | | EL vs RL | 0.53 | 0.1728 | 13090.14 | 3.07 | p = 0.0021 | 0.08 |
|  | LR  Non-ASD | | 18 | | EL vs FM | -0.33 | 0.2714 | 13181.79 | -1.20 | p = 0.2295 | 0.09 |
|  |  |  |  | | RL vs FM | 2.03 | 0.2655 | 13342.78 | 7.64 | p < .0004* | 0.57 |
|  |  |  |  | | VR vs FM | 0.50 | 0.2651 | 13337.63 | 1.89 | p = 0.0593 | 0.14 |
|  |  |  |  | | EL vs VR | -0.83 | 0.2612 | 13294.00 | -3.16 | p = 0.0016 | 0.24 |
|  |  |  |  | | RL vs VR | 1.53 | 0.2535 | 13105.44 | 6.02 | p < .0004* | 0.43 |
|  |  |  |  | | EL vs RL | -2.35 | 0.2615 | 13290.16 | -9.00 | p < .0004* | 0.67 |
|  |  | | 24 | | EL vs FM | 0.62 | 0.1872 | 13169.57 | 3.32 | p = 0.0009 | 0.14 |
|  |  | |  | | RL vs FM | 2.12 | 0.1837 | 13310.99 | 11.52 | p < .0004* | 0.46 |
|  |  | |  | | VR vs FM | 2.09 | 0.1835 | 13304.91 | 11.36 | p < .0004* | 0.45 |
|  |  | |  | | EL vs VR | -1.46 | 0.1810 | 13260.91 | -8.09 | p < .0004* | 0.32 |
|  |  | |  | | RL vs VR | 0.03 | 0.1765 | 13100.90 | 0.17 | p = 0.8626 | 0.01 |
|  |  | |  | | EL vs RL | -1.49 | 0.1812 | 13260.36 | -8.25 | p < .0004* | 0.32 |
|  |  | | 36 | | EL vs FM | 2.52 | 0.2237 | 13092.33 | 11.25 | p < .0004* | 0.38 |
|  |  | |  | | RL vs FM | 2.29 | 0.2236 | 13092.53 | 10.26 | p < .0004* | 0.34 |
|  |  | |  | | VR vs FM | 5.26 | 0.2232 | 13085.44 | 23.55 | p < .0004* | 0.78 |
|  |  | |  | | EL vs VR | -2.74 | 0.2232 | 13087.41 | -12.27 | p < .0004* | 0.41 |
|  |  | |  | | RL vs VR | -2.96 | 0.2231 | 13084.09 | -13.28 | p < .0004* | 0.44 |
|  |  | |  | | EL vs RL | 0.22 | 0.2235 | 13083.17 | 1.00 | p = 0.3180 | 0.03 |

^a^Significance using Bonferroni correction for alpha (.05/120).

^b^EL=Expressive Language; RL=Receptive Language; FM=Fine Motor; VR=Visual Reception.
