# Supplementary material for: Early sex differences are not autism-specific: A Baby Siblings Research Consortium (BSRC) study
Source: Mol Autism. 2015 Jun 4;6:32. doi: 10.1186/s13229-015-0027-y (PMC4455973; doi:10.1186/s13229-015-0027-y)
Supplement: Additional file 5: Table S5. — ADOS Domain estimated marginal means. Estimated marginal means and standard error for ADOS domains (RRB, SA) for each group by sex by age. [file 13229_2015_27_MOESM5_ESM.docx]

Table S5: ADOS Domain Estimated Marginal Means.

| **Domain** | **Group** | **Sex** | **Age** | **Estimate** | **SE** | **Lower Bound** | **Upper Bound** |
| --- | --- | --- | --- | --- | --- | --- | --- |
| Repetitive Behavior | ASD | Female | 24 | 7.07 | 0.35 | 6.43 | 7.79 |
|  |  |  | 36 | 6.94 | 0.57 | 5.92 | 8.16 |
|  |  | Male | 24 | 7.06 | 0.19 | 6.69 | 7.44 |
|  |  |  | 36 | 7.74 | 0.21 | 7.34 | 8.17 |
|  | HR  Non-ASD | Female | 24 | 4.23 | 0.15 | 3.94 | 4.54 |
|  |  |  | 36 | 3.50 | 0.47 | 2.73 | 4.61 |
|  |  | Male | 24 | 4.66 | 0.15 | 4.38 | 4.96 |
|  |  |  | 36 | 4.89 | 0.29 | 4.35 | 5.51 |
|  | LR  Non-ASD | Female | 24 | 3.24 | 0.20 | 2.89 | 3.66 |
|  |  |  | 36 | 2.26 | 0.81 | 1.36 | 5.43 |
|  |  | Male | 24 | 3.67 | 0.18 | 3.34 | 4.04 |
|  |  |  | 36 | 3.06 | 0.60 | 2.16 | 4.65 |
| Social Affect | ASD | Female | 24 | 5.74 | 0.41 | 5.01 | 6.61 |
|  |  |  | 36 | 6.54 | 0.47 | 5.70 | 7.54 |
|  |  | Male | 24 | 4.97 | 0.18 | 4.63 | 5.34 |
|  |  |  | 36 | 6.44 | 0.20 | 6.05 | 6.85 |
|  | HR  Non-ASD | Female | 24 | 2.39 | 0.09 | 2.22 | 2.59 |
|  |  |  | 36 | 2.24 | 0.23 | 1.87 | 2.78 |
|  |  | Male | 24 | 2.52 | 0.09 | 2.34 | 2.71 |
|  |  |  | 36 | 2.13 | 0.19 | 1.82 | 2.56 |
|  | LR  Non-ASD | Female | 24 | 1.69 | 0.09 | 1.54 | 1.88 |
|  |  |  | 36 | 2.86 | 0.28 | 2.39 | 3.48 |
|  |  | Male | 24 | 1.85 | 0.09 | 1.70 | 2.05 |
|  |  |  | 36 | 2.28 | 0.49 | 1.61 | 3.71 |
