# Supplementary material for: Early sex differences are not autism-specific: A Baby Siblings Research Consortium (BSRC) study
Source: Mol Autism. 2015 Jun 4;6:32. doi: 10.1186/s13229-015-0027-y (PMC4455973; doi:10.1186/s13229-015-0027-y)
Supplement: Additional file 6: Table S6. — ADOS Sex by Domain simple effects. ADOS domain comparisons by sex. [file 13229_2015_27_MOESM6_ESM.docx]

Table S6: ADOS Sex by Domain simple effects.

| **Comparison** | **Difference** | **SE** | **t-value** | **p-value** | **Effect (d)** |
| --- | --- | --- | --- | --- | --- |
| Female RRB - Female SA | 0.94 | 0.34 | 2.76 | p < .01* | 0.40 |
| Female RRB - Male RRB | -0.81 | 0.38 | -2.11 | p < .05* | 0.29 |
| Female SA - Male SA | 0.14 | 0.17 | 0.80 | p = .429 | 0.07 |
| Male RRB - Male SA | 1.89 | 0.20 | 9.36 | p < .001* | 0.71 |
