# Supplementary material for: Early sex differences are not autism-specific: A Baby Siblings Research Consortium (BSRC) study
Source: Mol Autism. 2015 Jun 4;6:32. doi: 10.1186/s13229-015-0027-y (PMC4455973; doi:10.1186/s13229-015-0027-y)
Supplement: Additional file 7: Table S7. — ADOS Group by Domain by Age simple effects. Comparisons by group, age, and ADOS domain. [file 13229_2015_27_MOESM7_ESM.docx]

Table S7: ADOS Group by Domain by Age simple effects.

| **Domain comparisons within Group and Age** | | | | | | | | |
| --- | --- | --- | --- | --- | --- | --- | --- | --- |
|  | **Group** | **Age** | **Comparison** | **Difference** | **SE** | **t-value** | **p-value^a^** | **Effect (d)** |
|  | ASD | 24 | RRB^b^ vs SA | 1.53 | 0.23 | 6.77 | p < .001* | 0.63 |
|  |  | 36 | RRB vs SA | 0.66 | 0.377 | 1.00 | p = .081* | 0.35 |
|  | HR  Non-ASD | 24 | RRB vs SA | 1.85 | 0.11 | 16.52 | p < .001* | 0.83 |
|  |  | 36 | RRB vs SA | 1.77 | 0.32 | 5.53 | p < .001* | 0.96 |
|  | LR  Non-ASD | 24 | RRB vs SA | 1.72 | 0.15 | 11.62 | p < .001* | 0.93 |
|  |  | 36 | RRB vs SA | -0.10 | 0.62 | -0.16 | p = .876 | 0.08 |
| **Age comparisons within Group and Domain** | | | | | | | | |
|  | **Group** | **Domain** | **Comparison** | **Difference** | **SE** | **t-value** | **p-value** | **Effect (d)** |
|  | ASD | RRB | 24 vs 36 months | -0.46 | 0.39 | -1.17 | p = .240 | 0.21 |
|  |  | SA | 24 vs 36 months | -1.34 | 0.345 | 1.00 | p < .001* | 0.60 |
|  | HR  Non-ASD | RRB | 24 vs 36 months | 0.27 | 0.31 | 0.86 | p = .381 | 0.11 |
|  |  | SA | 24 vs 36 months | 0.20 | 0.15 | 1.35 | p = .183 | 0.13 |
|  | LR  Non-ASD | RRB | 24 vs 36 months | 0.95 | 0.54 | 1.78 | p = .076 | 0.40 |
|  |  | SA | 24 vs 36 months | -0.87 | 0.36 | -2.39 | p = .017 | 0.78 |
| **Group comparisons within Domain and Age** | | | | | | | | |
|  | **Domain** | **Age** | **Comparison** | **Difference** | **SE** | **t-value** | **p-value** | **Effect (d)** |
|  | RRB | 24 | ASD vs HR Non-ASD | 2.25 | 0.23 | 9.96 | p < .001* | 0.82 |
|  |  |  | ASD vs LR Non-ASD | 2.90 | 0.26 | 11.20 | p < .001* | 1.05 |
|  |  |  | HR Non-ASD vs LR Non-ASD | 0.65 | 0.18 | 3.65 | p < .001* | 0.24 |
|  |  | 36 | ASD vs HR Non-ASD | 2.99 | 0.45 | 6.64 | p < .001* | 1.04 |
|  |  |  | ASD vs LR Non-ASD | 4.31 | 0.64 | 6.74 | p < .001* | 1.50 |
|  |  |  | HR Non-ASD vs LR Non-ASD | 1.33 | 0.61 | 2.17 | p = .031 | 0.46 |
|  | SA | 24 | ASD vs HR Non-ASD | 2.57 | 0.22 | 11.63 | p < .001* | 1.31 |
|  |  |  | ASD vs LR Non-ASD | 3.09 | 0.23 | 13.49 | p < .001* | 1.57 |
|  |  |  | HR Non-ASD vs LR Non-ASD | 0.52 | 0.09 | 5.84 | p < .001* | 0.26 |
|  |  | 36 | ASD vs HR Non-ASD | 4.10 | 0.34 | 11.95 | p < .001* | 1.51 |
|  |  |  | ASD vs LR Non-ASD | 3.56 | 0.48 | 7.45 | p < .001* | 1.31 |
|  |  |  | HR Non-ASD vs LR Non-ASD | -0.54 | 0.39 | -1.40 | p = .161 | 0.20 |

^a^Significance using Bonferroni correction for alpha (.05/24).

^b^RRB=Restricted and Repetitive Behavior; SA=Social Affective.
